# The perceptions and attitudes of patients with epilepsy to the use of a seizure diary, South Africa

**DOI:** 10.4102/safp.v65i1.5503

**Published:** 2023-01-09

**Authors:** Chika K. Egenasi, Anandan A. Moodley, Wilhelm J. Steinberg, Gina Joubert

**Affiliations:** 1 Department of Family Medicine, School of Clinical Medicine, Faculty of Health Sciences, University of the Free State, Bloemfontein, South Africa; 2 Department of Neurology, University of KwaZulu-Natal, Durban, South Africa; 3 Department of Biostatistics, School of Biomedical Sciences, Faculty of Health Sciences, University of the Free State, Bloemfontein, South Africa

**Keywords:** seizure diary, epilepsy, paper diary, electronic diary, seizure frequency, patients who had previous exposure to the seizure diary, unexposed

## Abstract

**Background:**

Epilepsy is responsible for a significant proportion of the world’s disease burden, affecting around 50 million people globally. A seizure diary is a self-management tool for epilepsy focusing on self-monitoring, tracking seizures and other symptoms. This study aimed to determine the perceptions and attitudes to the seizure diary in patients with epilepsy in the Free State and Northern Cape of South Africa.

**Methods:**

This cross-sectional survey method included adult patients with epilepsy attending Universitas Academic Hospital Specialist Epilepsy Clinic in Bloemfontein and local clinics in Kimberley (City, Beaconsfield and Betty Gatsewe), as well as the casualty department of Kimberley hospital (Robert Mangaliso Sobukwe Hospital). The Kimberley patients were diary-unexposed, while the Bloemfontein patients were patients who had previous exposure to the seizure diary.

**Results:**

A total of 182 patients with epilepsy were recruited for the study, of whom 65 were patients who had previous exposure to the seizure diary, and 117 were unexposed. In the patients who had previous exposure to the seizure diary, 64 (98.5%) found the diary useful, but 15 (23.1%) reported having various challenges with using the seizure diary. Almost all of the patients who had previous exposure to the seizure diary, 64 (98.5%), were willing to continue to use the diary, while 112 (95.7%) of the diary-unexposed patients were also willing to use the diary.

**Conclusion:**

Information from some patients using the diary confirms various challenges with its use; however, most patients support the continued usage of the diary.

## Introduction

The World Health Organization (WHO) estimates that epilepsy affects about 50 million people worldwide, of which about 10 million reside in Africa.^1^ In South Africa, epilepsy affects about 500 000 people.^2^ The WHO also estimates that about 100 million people will have a seizure at some point at least once in their lifetime;^1^ about 50 million will have recurrent seizures, of which 40 million will go untreated.^1^ About 70% of people with epilepsy can live a seizure-free life if resources are readily available to treat them.^1^ The WHO estimates that 80% of the 10 million patients with epilepsy residing in Africa will go untreated despite the availability of effective medications.^1^ Management aims to control the seizures to reduce morbidity and mortality associated with epilepsy.^3^

A diary is a tool meant to capture patients’ experiences close to the point of occurrence in order to provide more accurate data.^4^ A seizure diary is a self-management tool for epilepsy focusing on self-monitoring, tracking seizures and managing anti-seizure medications.^5^ It helps manage medication and other therapies, recognise triggers and health events that may affect seizures and wellness and communicate with the patient care providers.^6^ The seizure diary helps to capture the patients’ ‘life as it is lived’.^7^ The diary comes in two formats, the paper format and the electronic format. The paper diary is a basic calendar asking basic questions about patients’ seizure types and frequency, which is the industry standard.^8^ Patients can indicate the days of the month they had seizures by circling the dates in the calendar. On the other hand, the electronic diary is a digital format that can be uploaded online. It is a more detailed and accurate diary with time-stamped patient entries available to patients, caregivers and healthcare workers.

In countries such as the United States (US), Australia and Canada, various free online electronic seizure diaries are used, such as My Seizure Diary and Seizure Tracker.^9,10^

Many patients with epilepsy are already using them to help track seizure history, symptoms and medication management and help patients interact with their healthcare providers.^11^ Seizure Tracker is an online seizure diary with over 20 000 registrants tracking their seizures electronically.^12^ In a country like the US, patients have access to personal electronic devices;^13^ however, in impoverished communities in South Africa, mobile device usage is severely constrained due to the high cost.^14^ It also diverts income from more productive uses in resource-poor settings.^14^ Some people live in rural locations^15^ where Internet coverage is not always available, making it difficult for them to use electronic-based diaries. In such communities, the paper diary can be used, given sufficient reading and language skills.^16^ It does not require any Internet access or basic computer skills for the patients to use.^16^

In South Africa, there is no standardised format for the seizure diary. A calendar format of the diary is used in some South African medical facilities. Most of the scientific papers reviewed support the concept that a properly designed seizure diary has a role in managing epilepsy patients and improving medication adherence in the clinical setting.^17,18,19^

If modified for patient use with the active involvement of patients, the seizure diary can be a useful tool to help the patients and their relatives keep records of events related to epilepsy, such as seizures, in their own words. The diary may also help them relate their experiences so that the healthcare workers can understand how the disease impacts them and their families. The diary can help healthcare workers access essential information about their patients which otherwise may not have been available. The available information may eventually lead to better management of patients, with minimum wastage of scarce resources. By developing a seizure diary that can be effectively used to monitor and manage patients with epilepsy in South Africa, this study aimed to determine patients’ perceptions and attitudes to the use of the seizure diary in managing patients with epilepsy in the Free State and Northern Cape of South Africa.

## Methodology

### Study design

The study was part of a more extensive study conducted in phases. This study was Phase 2, a cross-sectional study. Other phases consisted of Phase 1 (scoping review of literature), Phase 3 (Delphi study), Phase 4 (introduction to the new, improved seizure diary), Phase 5a (patients’ perceptions of the new, improved seizure diary) and Phase 5b (suggested final version of the seizure diary).

### Study population

The study population consisted of adults with epilepsy in Kimberley and Bloemfontein currently attending Universitas Academic Hospital Specialist Epilepsy Clinic in Bloemfontein and local clinics in Kimberley (City, Beaconsfield and Betty Gatsewe), as well as the casualty department in Kimberley’s Robert Mangaliso Sobukwe Hospital from January 2021 to July 2021. The Bloemfontein population were previous diary users (exposed) to a basic calendar diary, while the Kimberley population were unexposed to the diary. Relatives and caregivers involved in taking care of the patients could help complete the questionnaire. Questionnaires completed by relatives or caregivers were noted as such.

### Inclusion criteria

Patients diagnosed with epilepsy by a medical practitioner attending the facility for follow-up and 18 years and above were recruited for the study. Patients with cognitive impairment were included, as relatives provided information. Recruitment sites included Universitas Academic Hospital Specialist Epilepsy Clinic in Bloemfontein, local clinics (City, Beaconsfield and Betty Gatsewe) and the casualty department in Kimberley’s Robert Mangaliso Sobukwe Hospital. Informed consent was obtained from patients or caregivers when patients were disabled owing to cognitive impairment.

### Exclusion criteria

Patients with seizures not diagnosed as epilepsy were excluded.

### Sampling method

Consecutive sampling was used in selecting patients meeting the inclusion criteria, depending on patients’ availability and willingness to be enrolled in the study. Patients were enrolled in the study until the required sample size was attained.

### Sample size

Given the number of patients available, it was decided to include 80 patients in each group. Patients who had previous exposure to the seizure diary from Universitas Academic Hospital epilepsy specialist clinics versus diary-unexposed patients from the Kimberley hospital (Robert Mangaliso Sobukwe Hospital) casualty department, City, Beaconsfield and Betty Gatsewe clinics. Thus, no formal sample size calculation was performed, and this study had no main outcome on which to base such a calculation, but this group size gives sufficient power (80%) to detect large differences between the groups (e.g. 60% vs. 80%) and fairly precise estimates within groups.

### Measurement

A confidential, structured questionnaire with 33 questions was administered to the study patients in the Bloemfontein and Kimberley populations. The questionnaire was designed from information obtained from literature on the use of the seizure diary after an extensive literature search. It consisted of five sections, A–E. Section A consisted of seven demographic questions, mostly requiring ‘Yes’ or ‘No’ answers, and one question on patients’ location. Section B had two questions about the patient’s epilepsy. Section C consisted of six questions about the patient’s seizures. Five questions required the patient to choose the most appropriate option for them, and one question required a ‘Yes’ or ‘No’ answer. Section D had 16 questions, 14 of which required a ‘Yes’ or ‘No’ answer, and two open-ended questions.

The initial two questions asked the patients if they had ever heard or used the diary; if the response to using the diary was ‘No’, the patient was then requested to move on to Section E. If the response to the use of the diary was ‘Yes’, the patient was then requested to answer the rest of the 14 questions on the use of the seizure diary. Section E had two questions about the future use of the diary, requiring a ‘Yes’ or ‘No’ answer. The same questionnaire was used for both populations. The questionnaires were available in English, Afrikaans, Setswana and Sesotho. Two research assistants with healthcare experience were trained to assist the researcher. They were required to assist with studying patients only when off duty. They were able to communicate in English, Afrikaans, Setswana or Sesotho; and they were willing to work with patients with epilepsy and ready to travel to different research sites in Kimberley and Bloemfontein.

The questionnaires were manually distributed by the first author or the assistants in City, Betty Gatsewe and Beaconsfield clinics and Kimberley hospital (Robert Mangaliso Sobukwe Hospital) casualty while patients were in the waiting area. In Bloemfontein, questionnaires were distributed to patients attending the neurology clinic while waiting to be called into the consulting room by the doctor to prevent wasting the patients’ time after consultation. The first author and his assistants individually explained the process and obtained informed consent from the patients. All patients were required to immediately complete the questionnaires and return the completed questionnaire to the researcher or his assistants while still in the waiting area. Illiterate patients were assisted by their relatives, caregivers, the researcher or trained assistants fluent in one of the spoken languages. Cognitively impaired patients were assisted by their relatives or caregivers. The first author and his assistants also used the opportunity to educate diary unexposed patients, their relatives and caregivers about the seizure diary.

### Pilot study

A pilot study was done on epilepsy patients in Kimberley hospital casualty and Universitas Academic Hospital neurology specialist clinic. It included five patients who met the inclusion criteria. This tested the questionnaire and the project processes. Data from the pilot study was not used in the main study because of changes made in the questionnaire.

### Statistical analysis

Categorical variables were summarised by frequencies and percentages and numerical variables by median and interquartile ranges (IQRs) because of skewed distributions. Groups were compared regarding categorical variables using chi-square or Fisher’s exact tests (in the case of sparse cells). The significance level was set at 0.05. Analysis was performed using SAS version 9.4.

### Ethical considerations

The protocol for the study was approved by the Health Sciences Research Ethics committee (HSREC) of the University of the Free State (re. no. UFS-HSD2020/1385/2411). Approval for data collection was also obtained from the Northern Cape Health Department and the Free State Health Department.

Number coding was used to ensure the confidentiality of the participant’s responses. No names or personal identifiers appeared on any research-related information or datasheet sent for statistical analysis. All paper-based records were kept in a secure location by the researcher and were only accessible to those involved in the study. All information was managed in a strictly professional and confidential manner.

## Results

A total of 186 patients were recruited for the study from January 2021 to July 2021 from Universitas specialist hospital in Bloemfontein and the Kimberley casualty department and clinics. A final sample size of 182 was obtained after four patients were excluded for incomplete questionnaires. Only 65 patients who had previous exposure to the seizure diary were available for recruitment and 117 unexposed. More patients than the initially intended 160 patients were recruited to compensate for possible dropouts in-between phases.

The median age was 31 (range 18–76 years) in the patients who had previous exposure to the seizure diary and 38 (range 18–68 years) in the unexposed group. The *p*-values for age were < 0.01, 95% confidence interval (CI) for median difference patients who had previous exposure to a seizure diary versus not exposed, –10; –2.

Table 1 summarises patients’ demographic data. There were significantly more women and pensioners in the patients who had previous exposure to the seizure diary group, and a large percentage of patients in the diary unexposed group had below Grade 12 education.

**TABLE 1:** Demographic characteristics.

Variable	Patients who had previous exposure to the seizure diary (*n* = 65)		Diary-unexposed (*n* = 117)		*p*
	*n*	%	*n*	%	
**Gender**					< 0.01*
Male	25	38.5	75	64.1	
Female	40	61.5	42	35.9	
**Occupation**					0.03*
Employed	5	7.7	17	14.5	
Pensioner	35	53.9	42	35.9	
Student	5	7.7	4	3.4	
Unemployed	20	30.8	54	46.2	
**Education**					< 0.01*
Below Grade 12	37	56.9	72	61.5	
Matric	5	7.7	27	23.1	
Others	19	29.2	15	12.8	
University	4	6.2	3	2.6	

* , Statistically significant difference.

**FIGURE 1:**
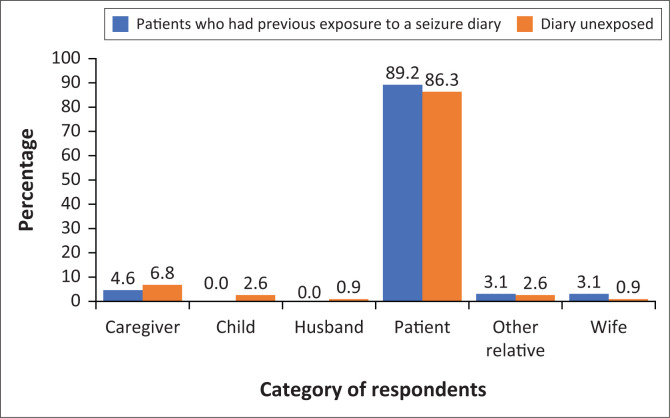
Persons completing the questionnaire.

Figure 1 shows the distribution of people completing the patient questionnaire. The majority of the patients who had previous exposure to the seizure diary and the unexposed patients completed the questionnaire themselves.

Table 2 illustrates patients’ reported responses regarding their disease. The disease duration of patients who had previous exposure to the seizure diary was significantly longer. The patients who had previous exposure to the seizure diary group had a significantly larger percentage with unknown seizure types, and the unexposed group had a significantly larger percentage reporting generalised tonic-clonic seizures.

**TABLE 2:** Disease characteristics.

Variable	Patients who had previous exposure to a seizure diary (%)	Diary unexposed (%)	*p*
**Disease duration (in years)**			0.04*
Less than 1	0.0	11.1	
1–5	16.9	19.7	
6–10	16.9	12.8	
More than 11	66.1	56.4	
**Types of seizure**			< 0.01 *
Absence seizures	9.2	6.8	
Focal seizures	0.0	7.7	
Generalised tonic-clonic	6.2	27.4	
Don’t know	86.4	57.3	
Others	0.0	0.9	
**Annual seizure frequency**			0.77
None	7.7	9.4	
Once	9.2	12.0	
More than once	83.1	78.6	
**Treatment with anti-epilepsy drugs**			0.09
No	0.0	5.1	
Yes	100.0	94.9	

* , Statistically significant difference.

Table 3 reports patients’ responses to questions about seizure awareness and recall of seizures.

**TABLE 3:** Awareness and recall of seizures.

Questions	Patients who had previous exposure to the seizure diary						Diary unexposed						*p*
	Always		Sometimes		Never		Always		Sometimes		Never		
	*n*	%	*n*	%	*n*	%	*n*	%	*n*	%	*n*	%	
Are you able to remember you had a fit after an episode?	15	23.1	16	24.6	34	52.3	24	20.5	47	40.2	46	39.3	0.10
Do you need someone to inform you that you had a fit?	40	61.5	8	12.3	17	26.2	60	51.3	44	37.6	13	11.1	< 0.01 *
Are you able to recall how many seizures you have in a month?	28	43.1	21	32.3	16	24.6	25	21.4	51	43.6	41	35.0	0.01 *

* , Statistically significant differences.

A few diary-unexposed patients (6.8%) have heard of the seizure diary. When asked whether they knew the diary was free, 63 (96.9%) of the patients who had previous exposure to the seizure diary knew it was free. Almost all 64 (98.5%) of the patients who had previous exposure to the seizure diary were willing to continue to use the diary, while 112 (95.7%) of the diary-unexposed group were willing to use the diary (*p* = 0.42). All our patients who had previous exposure to the seizure diary were on anti-epileptic treatment except for one omission, while 111 (94.9%) of the diary-unexposed patients reported being on treatment with anti-epileptic medications.

Of all the patients using the diary, 65 (100.0%) were recommended it by their doctors, 64 (98.5%) reported that they understood the diary and 56 (86.2%) used the diary every time they had seizures. Almost all 62 (95.4%) liked using the diary, 64 (98.6%) of patients who had previous exposure to the seizure diary found the diary helpful in monitoring their epilepsy and 65 (100.0%) reported that the diary helped them keep track of their seizures and how often they had seizures. Of the patients using the diary, 34 (52.3%) did not find the diary helpful in taking their medications compared to 31 (47.7%) who found it useful for that purpose. Most patients, 50 (76.9%), did not report any challenges with completing the diary, while 15 (23.1%) reported some challenges, as described in Table 4.

**TABLE 4:** Challenges with keeping a seizure diary.

Challenges	*n*	%
Don’t usually remember to complete it	6	9.2
It is not important to me	4	6.2
No time to complete the diary	3	4.6
Not trained to use the diary	3	4.6
Don’t find the diary useful	3	4.6
Not motivated to complete it	3	4.6
It is difficult to complete	3	4.6
Having to complete the diary makes me tired	3	4.6
Nobody to help them complete the diary	2	3.1
Diary got lost	2	3.1
Doctor does not check the diary	1	1.5
It reminds me of my condition	1	1.5

When patients who had previous exposure to the seizure diary were given multiple options to choose from about what information they think is essential to be in the diary, 48 (73.9%) requested the date of the seizure, 25 (38.5%) frequency of the seizure, 35 (53.9%) asked for the type of the seizure to be included. Medication and dosages were only chosen by 17 (26.2%) of patients, 21 (32.3%) emergency contact details, 15 (23.1%) doctors details. Only one patient (1.5%) requested space on the diary for more than one seizure per day. Almost all of the patients who had previous exposure to the seizure diary, 63 (98.4%), felt it was important to hand the diary over to their healthcare providers during each visit.

## Discussion

The study investigated patients’ perceptions and attitudes to the seizure diary in Kimberley and Bloemfontein. Uncontrolled epilepsy with seizures of more than one a year was reported by more of the patients who had previous exposure to the seizure diary than the diary-unexposed patients. A possible explanation may be that patients who had previous exposure to the seizure diary attending the Universitas specialist hospital have more complex seizures with a higher seizure burden. They are more likely to monitor and document their seizures than patients with less complex seizures and a lower seizure burden. Detyniecki et al.^20^ reported in a study on patients with epilepsy that patients with a higher seizure burden are more likely to report their seizure activities.

Patients generally did not have a good knowledge of their epilepsy diagnosis. However, the diary-unexposed group had a better understanding of their type of seizure diagnosis than the patients who had previous exposure to the diary group. This may be because of the rigorous and extensive investigation required to diagnose the complicated situations of patients who had previous exposure to the seizure diary. The complicated syndromes may cause them to have less understanding. It can also be that the doctors, because of the complex nature of their diagnosis, may not have the time to inform, educate and ensure that patients with complicated epilepsy understand their diagnosis. A study done among cancer patients in Turkey revealed that a high number of patients, 44%, did not know their diagnosis, which did not worsen their quality of life.^21^ In another study from Cuba, patients aware of their diagnosis had significantly better scores with respect to symptoms, patient anxiety, information and support than patients not aware of their diagnosis.^22^ It is expected that patients with epilepsy who are aware of their diagnosis will be better informed about managing their medical condition and accessing available support.

Most patients in both groups had problems remembering if they had a seizure after an episode. These findings are supported by reports from other authors, such as Blachut et al.^23^ and Poochikian-Sarkissian et al.,^24^ who stated that a significant number of seizures go unnoticed by patients. If a patient cannot remember having a seizure, they will certainly not document it.

Many patients from both groups needed to be informed when they had a seizure, although more patients in the diary-unexposed group than those who had previous exposure to the seizure diary group needed to be informed about a seizure. Blachut et al.^23^ stated that 28% of patients required a seizure witness to remind them that a seizure occurred. Blum et al.^25^ reported that 30% of patients in a study were never aware of their seizures. This is in keeping with the report from this study’s patients, who reported that they needed to be informed about their seizures. The patients may not be aware of their seizures, more so with nocturnal seizures than daytime seizures; this was reported by several past studies.^13,23,26,27^

A more significant percentage of patients reported being able to recall their seizures in the patients who had previous exposure to the seizure diary group than the diary-unexposed groups. This may be because of the patients who had previous exposure to the seizure diary group being able to keep track of their seizure frequencies to complete their diaries. Some authors found seizure recall among patients with epilepsy to be unreliable.^23,24,25,27^

Most of the patients in both groups agreed that documenting their seizures would help them remember they had a seizure; it is expected that documenting their seizures will help patients keep track of the trend of their seizures. However, Hoppe et al.^27^ reported in their study that only a few patients were able to document most of their seizures accurately; a significant part of their seizures were undocumented. The accuracy of patient-documented seizures affects the validity of patient-reported seizures, as most patient reports are seen as subjective, requiring objective methods such as ambulatory electroencephalogram (EEG) to validate the accuracy of the information provided by patients.^23,24,27,28,29^ In the South African environment, this is unlikely to be suitable, as the affordability of the equipment is a challenge in a country with a significant disease burden that will make it unsustainable in the long run.

It was encouraging to see that almost all the patients who had previous exposure to the seizure diary and unexposed groups were willing to use or continue to use the diary. This shows a high level of acceptance of the seizure diary among the patients involved in the study. Schülin et al.,^30^ in a study on experiences with the use of pain diaries in chronic pain management, had a similar finding where most of the patients participating in the study were willing and able to use the pain diary and kept it voluntarily. More than half of the present study’s participants in the diary-exposed group did not find the diary useful in helping with their medication adherence. This is different from findings reported by Henegouwen et al.,^17^ suggesting that completing a daily diary is positively correlated with patient compliance with medication intake. Most of the current study’s patients already using the diary found it to be important and useful in their lives; this report was similar to findings from other literature.^23,30^

A few patients who had previous exposure to the seizure diary had various challenges that discouraged them from using the diary; they ranged from doctors not checking the diary to the diary being a painful reminder of their medical conditions. Most of the patients in this study felt it was important to hand the diary over to the healthcare practitioner managing them. Doctors are encouraged to show interest in the patient’s seizure diary; many authors have reported that if the patient senses that it is important in treatment decisions, it enables them to use the diary, as well as patients being motivated to fill out the diary because of their doctors. A study on the use of a diary in an intensive care unit (ICU) setting was found to positively impact the patients, relatives and the doctors and nurses involved in patient care.^31^

Healthcare practitioners can also train patients and relatives on using the diary. Bingham et al.,^32^ in their study on patient-reported outcomes (PRO) in patients with rheumatoid arthritis, revealed that appropriate training to use the electronic diary was one of the factors associated with high compliance. Training patients on how to use the diary will ensure that they understand it and remain motivated to use it.

The diary is meant to be a tool to help patients manage their epilepsy; it is not meant to hurt a patient by acting as a constant reminder of a medical condition a patient would prefer to rather forget. Patients need to be encouraged to avoid the denial of their medical condition and rather see the diary as a tool that can help improve their conditions. Healthcare practitioners should find exciting ways to motivate patients to use the diary and discuss any challenges they have with the diary to prevent noncompliance.

### Limitations of the study

The emphasis of the study was on patients from Kimberley and Bloemfontein, so it may have been subject to selection bias. This may make it difficult to generalise the findings of the study. The method used to collect the data may have further subjected the study to bias since consecutive sampling was used. Patients were selected based on meeting the inclusion criteria until the required sample size was achieved, which may not represent the population. The difference between the two samples may have caused some bias. The number of patients who had previous exposure to the seizure diary available for the study was smaller than expected. The number of patients attending our clinics was also reduced because of the advent of covid infection; rather than come to the clinic monthly for medications, patients now receive monthly six medications to reduce the number of patients attending the clinic daily.

## Conclusion

Almost all the patients involved in the study are willing to use the seizure diary. Patients who had previous exposure to a seizure diary enjoyed using the diary and found it helpful in monitoring the trend of their epilepsy. Many patients from both groups needed to be informed when they have a seizure. Patients who had previous exposure to the diary recalled their seizures better than diary-unexposed patients. The findings from this study confirm patients’ support for the use of the paper diary in the management of patients living with epilepsy.
